# The use of robotic arms for individuals with severe upper-limb disabilities

**DOI:** 10.1186/s42466-025-00403-1

**Published:** 2025-09-26

**Authors:** Noémie Fortin-Bédard, Orthelo Léonel Gbètoho Atigossou, Véronique H. Flamand, Jason Bouffard, François Routhier

**Affiliations:** 1 Center for Interdisciplinary Research in Rehabilitation and Social Integration, Centre intégré universitaire de santé et de services sociaux de la Capitale-Nationale, Quebec, Canada; 2 School of Rehabilitation Sciences, Faculty of Medicine, Université Laval, Quebec, Canada; 3 Department of Kinesiology, Faculty of Medicine, Université Laval, Quebec, Canada

**Keywords:** Robotic arm, Assistive technology, Life habits, Satisfaction, Quality of life

## Abstract

This letter to the editor aims to comment on the article « *User expectations and experiences of an assistive robotic arm in amyotrophic lateral sclerosis: a multicenter observational study* » recently published by Spittel et al. (Neurol Res Pract, 6(1), 42).

Dear Editor,

We have read with great interest the article « *User expectations and experiences of an assistive robotic arm in amyotrophic lateral sclerosis: a multicenter observational study* » recently published by Spittel et al. [9]. This multicenter observational study explored the expectations toward the JACO robotic arm among 85 potential users and documented the experience of 14 users with severe motor deficits of the arms due to amyotrophic lateral sclerosis (ALS). The findings represent a valuable addition to the scientific literature on assistive technologies for individuals with disabilities, addressing expectations prior to the provision of the JACO arm, user experience, the overall utility of the arm, the frequency of use, user recommendations, and the reasons that some participants did not receive the robotic arm [9].

While these findings relate to the ALS population, we believe that the study failed to recognize the comprehensive literature on the JACO arm [1, 2, 5, 7, 8] and iARM/MANUS robotic arm [4, 6, 10] in populations with severe upper-limb disabilities, such as individuals with muscular dystrophy, spinal muscular atrophy, cerebral palsy, spinal cord injury, and their caregivers. Since multiple medical conditions can negatively and severely affect the use of upper extremities, the authors could have leveraged the existing body of literature, which provides valuable insights regarding the use of robotic arms in other populations that may translate to individuals with ALS.

More precisely, Spittel et al. [9] noted that future studies could explore how robotic arms enhance daily living functions and, consequently, the quality of life for individuals with ALS. In fact, previous studies conducted among individuals with severe upper-limb disabilities have addressed these research questions and, in our opinion, may provide support for the use of robotic arms by the ALS population, such as the JACO arm. The existing literature reported the positive psychosocial impacts of using robotic arms [2, 7, 8], as well as user satisfaction with the technology [2, 7, 8] and a positive perception of quality of life of users [4, 6, 7, 10]. Robotic arms were also found to facilitate the accomplishment of certain life habits [2, 4, 10] (e.g., personal care) and to enable users to engage in a greater variety of life habits categories [7, 8]. Additionally, studies reported that the use of robotic arms reduced perceived caregiver burden [2, 8]. A study using an economic model inferred that the use of the JACO arm could potentially reduce caregiving time by 41% [5]. Similarly, a study demonstrated that the use of the MANUS robotic arm could lead to an average reduction of 1.2 h of human assistance per day [6].

Discussing the existing findings related to robotics arms is essential to provide readers with a deeper understanding of their implications as well as to explore external validity in relation to previous findings [3]. In that regard, we have identified similarities in the use of the JACO arm in the results reported by Spittel et al. [9] and those of previous studies, despite the differences in diagnoses. For example, in two studies [4, 8], robotic arms were predominantly used for eating and drinking. Similarly, the study of Spittel et al. [9] has found that serving food and drinks was the most common use. Furthermore, Spittel et al., reported that users perceived their JACO arm use as safe and were satisfied with the device, which is consistent with previous studies conducted among individuals with muscular dystrophy [8].

In sum, we believe that the integration of the findings reported in the study of Spittel et al. [9] with the existing body of scientific literature will contribute to improving the understanding and reflections on robotics arm use by individuals with severe upper-limb disabilities.

## Data Availability

Not applicable.

## Abbreviations

ALS: Amyotrophic lateral sclerosis
